# Authors’ Response to Peer Reviews of “Impact of the COVID-19 Pandemic on the Mental Health of College Students in India: Cross-sectional Web-Based Study”

**DOI:** 10.2196/32954

**Published:** 2021-09-02

**Authors:** Amar Prashad Chaudhary, Narayan Sah Sonar, Jamuna TR, Moumita Banerjee, Shailesh Yadav

**Affiliations:** 1 Department of Pharmacy Practice Mallige College of Pharmacy Rajiv Gandhi University of Health Sciences Bangalore India; 2 Department of Pharmacology Mallige College of Pharmacy Rajiv Gandhi University of Health Sciences Bangalore India; 3 Department of Pharmaceutics Mallige College of Pharmacy Rajiv Gandhi University of Health Sciences Bangalore India

*This is the authors’ response to peer-review reports for “Impact of the COVID-19 Pandemic on the Mental Health of College Students in India: Cross-Sectional Web-Based Study”.*

## Round 1 Review

The responses and changes made to the manuscript in reply to the reviewers’ comments [1,2] are below:

### Major comments

1. The novelty of this study [3] is justified in the manuscript in detail.
2. The grammatical errors in the manuscript have been corrected.
3. The keywords have been corrected.
4. The *Introduction* part has been revised with all the relevant information provided during the data collection time in India.
5. The literature review for this study has been expanded with relevant scholarly articles.
6. The concurrent validity of the Fear of COVID-19 Scale with the Generalized Anxiety Disorder-7 scale and the Patient Health Questionnaire-9 have been compared with the available prior evidence.

### Minor comments

1. Footnotes have been provided for the abbreviations in the table.
2. Other correlation values have been added to Table 4 along with *P* values.
3. For very small *P* values, *P*<0.001 has been written.
4. For Tables 4 and 5, the format has been corrected with clarity and written as per journal guidelines.
5. Less scholarly journal articles have been removed and references are written as per guidelines.

## Round 2 Review

### Minor comment

* and *** to indicate significance levels in the Abstract have been removed.
2. GAD-7 is the Generalized Anxiety Disorder Scale and PHQ-9 is the Brief Patient Health Questionnaire; this is indicated clearly in the main text in addition to the footnotes of the table. Different terms for the two scales have been removed and corrected.
3. Articles about university students regarding their psychological distress have been cited more, including Pramukti et al [4].
4. The reference category for the categorical independent variable is indicated in Table 4.
